# Supplementary figures and images for: G2S3: A gene graph-based imputation method for single-cell RNA sequencing data
Source: PLoS Comput Biol. 2021 May 18;17(5):e1009029. doi: 10.1371/journal.pcbi.1009029 (PMC8189489; doi:10.1371/journal.pcbi.1009029)

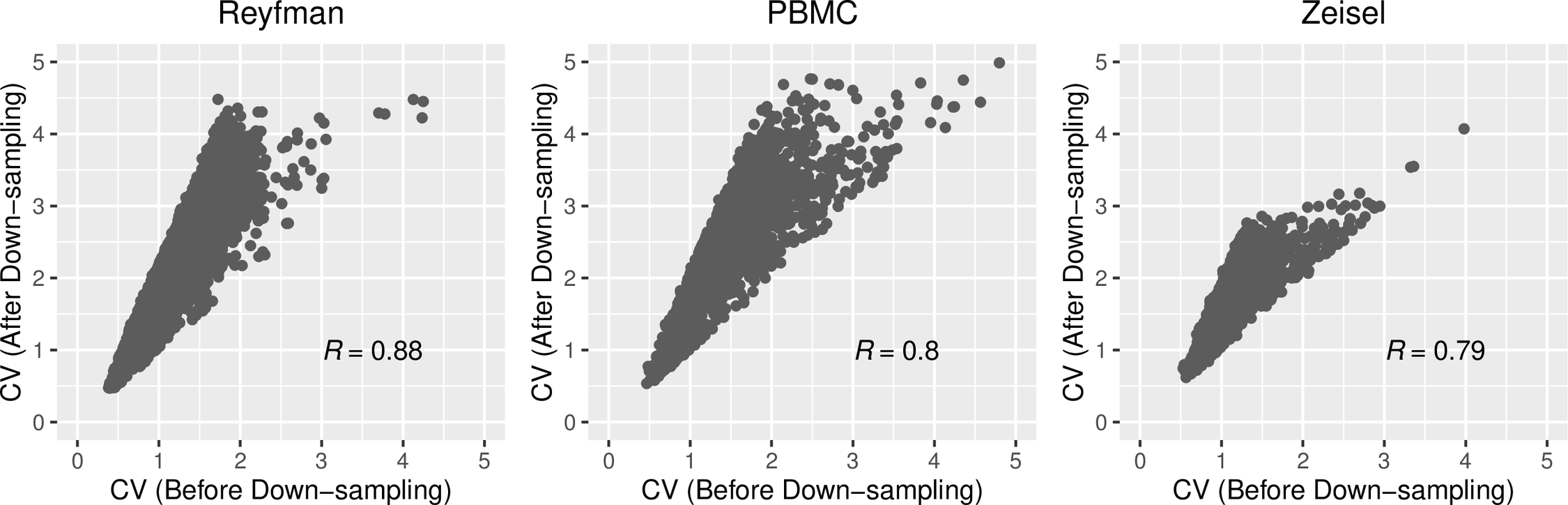

Supplement: S1 Fig — For each gene, the coefficient of variation (CV) across all cells after down-sampling (y-axis) is plotted against the CV of non-zero cells in the reference data (x-axis). (TIF) [file pcbi.1009029.s001.tif]

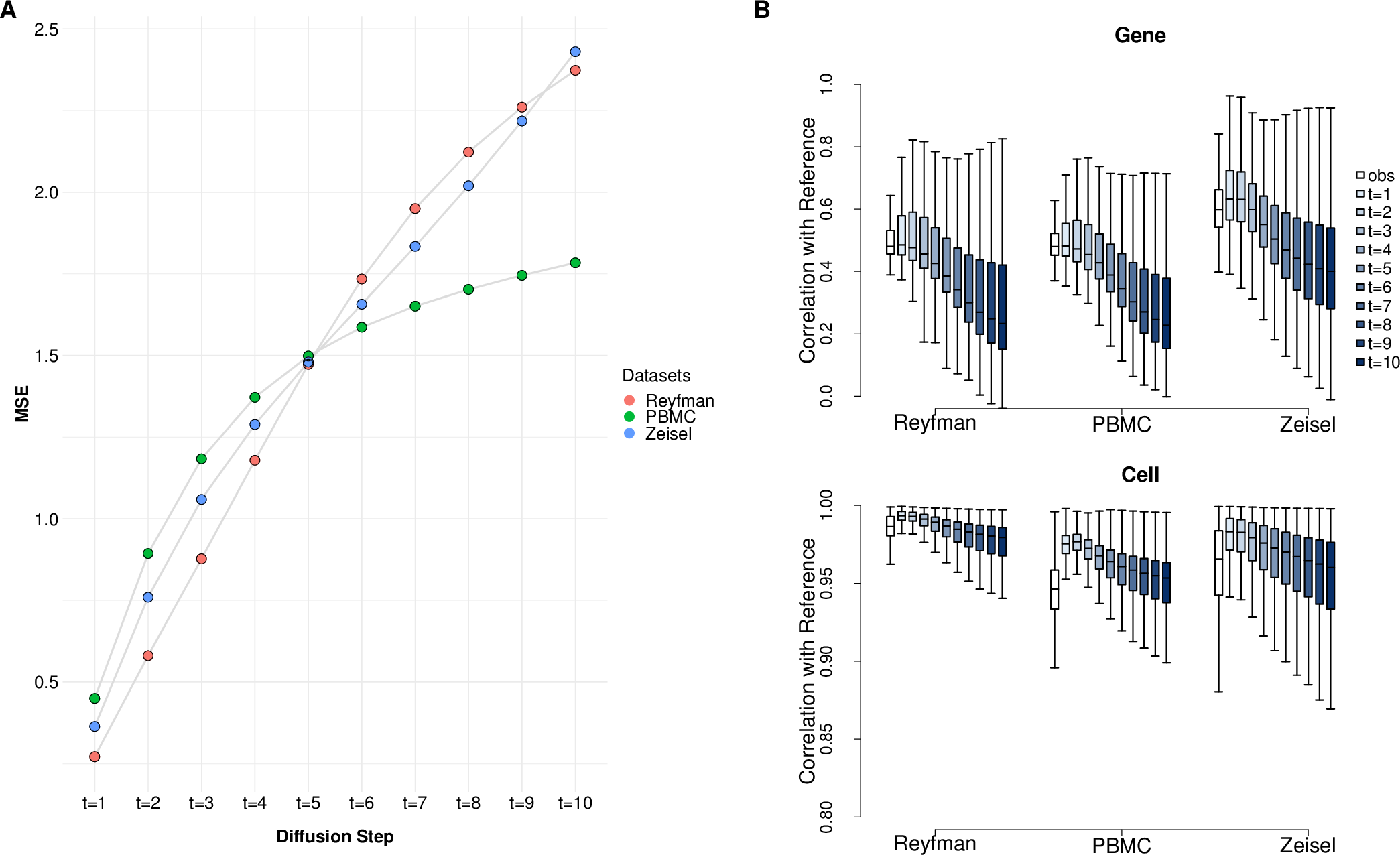

Supplement: S2 Fig — A. Mean squared error (MSE) at different diffusion steps in three down-sampled datasets. B. Gene-wise and cell-wise correlations of G2S3 imputed data at different diffusion steps and the reference data. (TIF) [file pcbi.1009029.s002.tif]

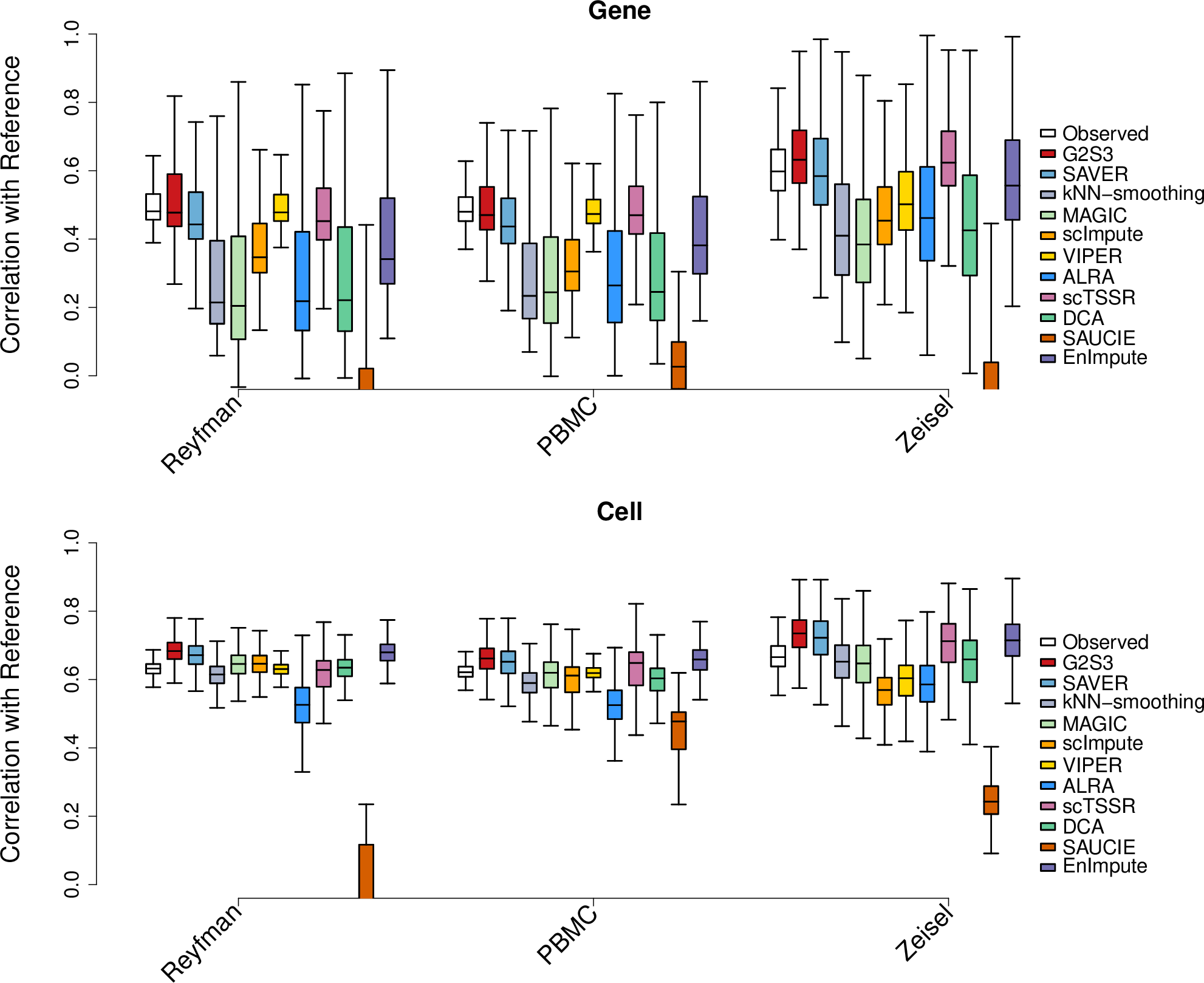

Supplement: S3 Fig — Performance of imputation methods measured by correlation with reference data from the first category of datasets, using gene-wise (top) and cell-wise (bottom) correlation. Box plots show the median (center line), interquartile range (hinges), and 1.5 times the interquartile (whiskers). (TIF) [file pcbi.1009029.s003.tif]

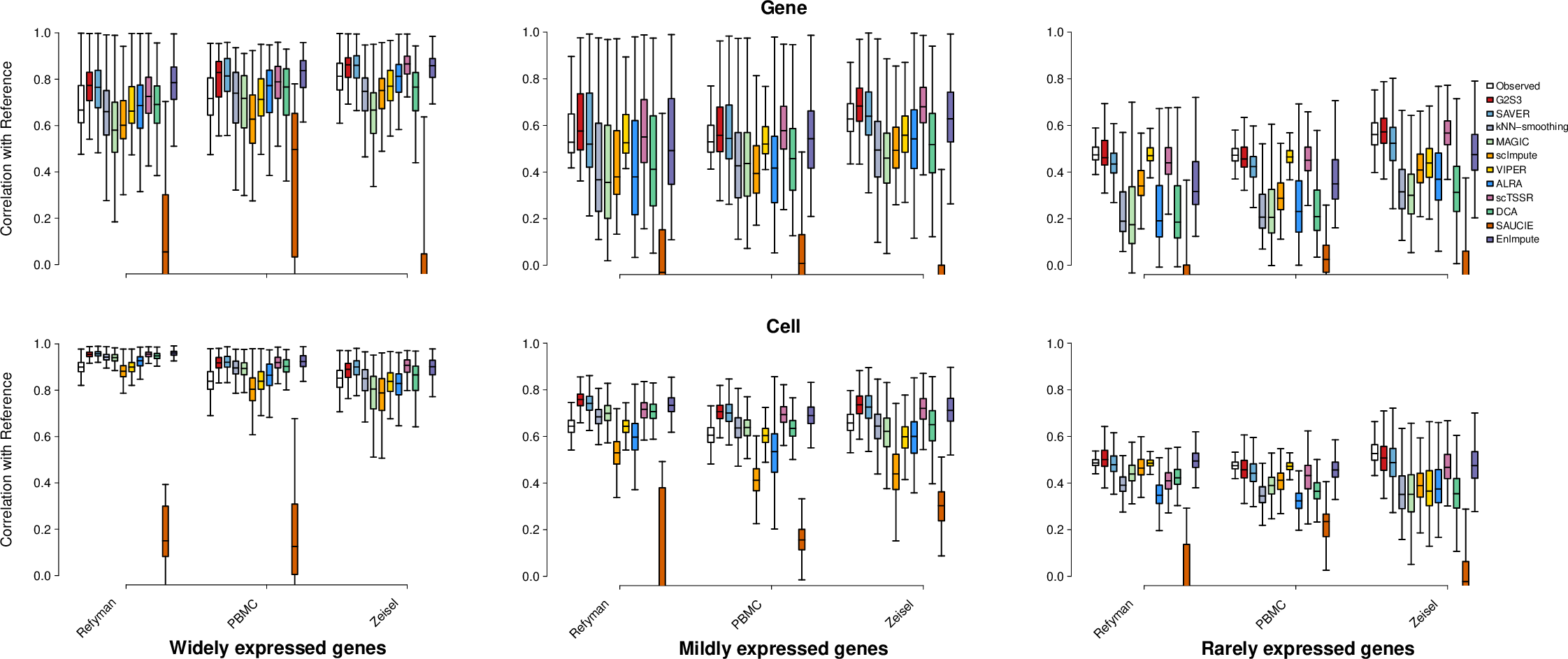

Supplement: S4 Fig — Performance of imputation methods measured by correlation with reference data from the first category of datasets, using gene-wise (top) and cell-wise (bottom) correlation. Genes are stratified into three groups: widely (>80%, left), mildly (30%-80%, middle), and rarely (<30%, right) expressed. (TIF) [file pcbi.1009029.s004.tif]

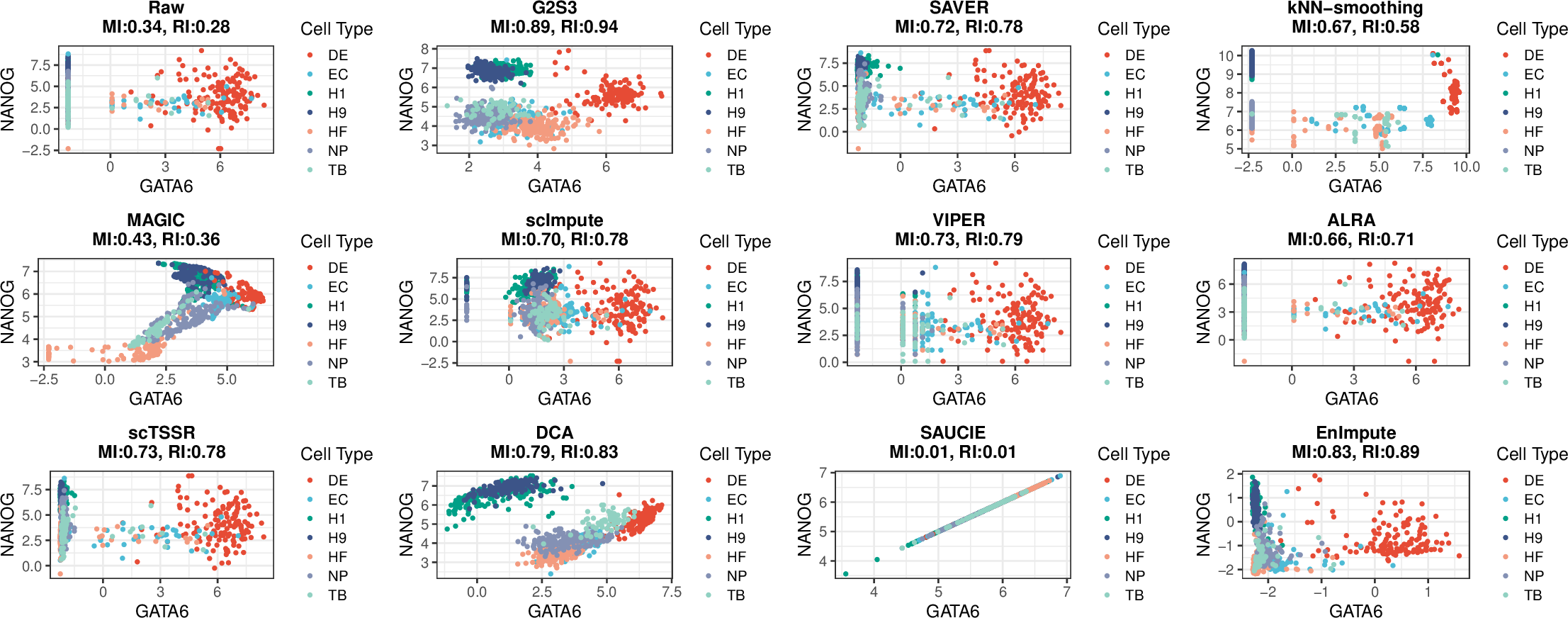

Supplement: S5 Fig — Scatter plot showing expression level of marker genes for DE cells (GATA6) and H1/H9 cells (NANOG). Cells are colored by the cell subtype labels. (TIF) [file pcbi.1009029.s005.tif]

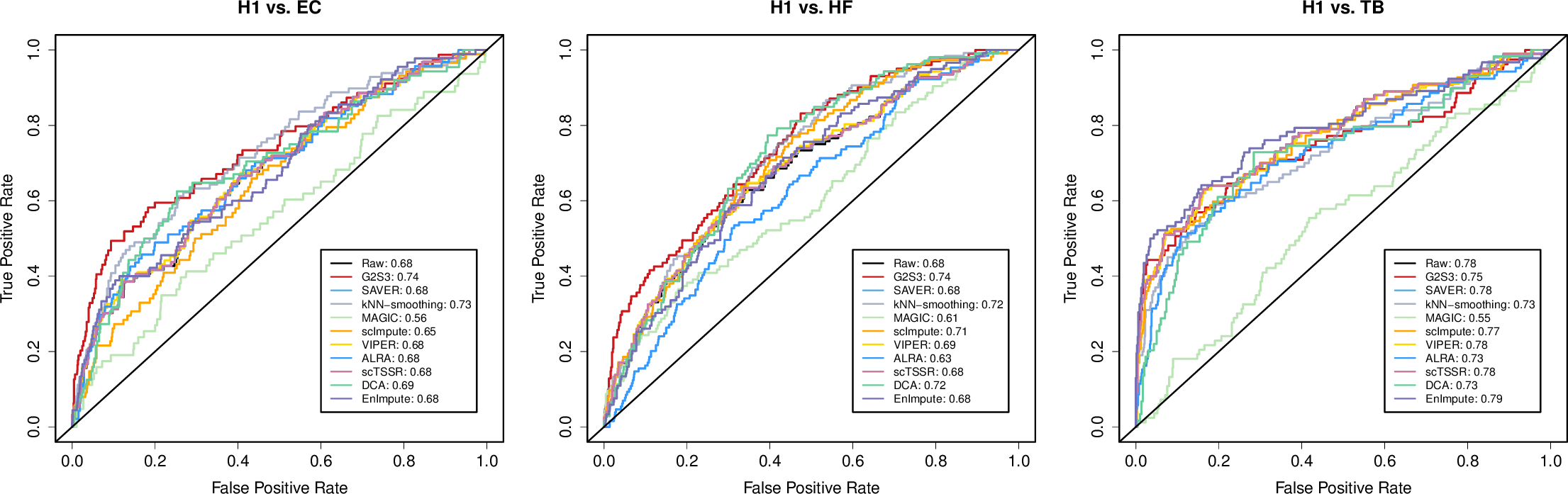

Supplement: S6 Fig — ROC curves measuring the prediction accuracy in scRNA-seq data on differentially expressed genes identified in bulk RNA-seq data comparing H1 to other homogeneous cell types (H1 vs. EC, H1 vs. HF, and H1 vs. TB). (TIF) [file pcbi.1009029.s006.tif]

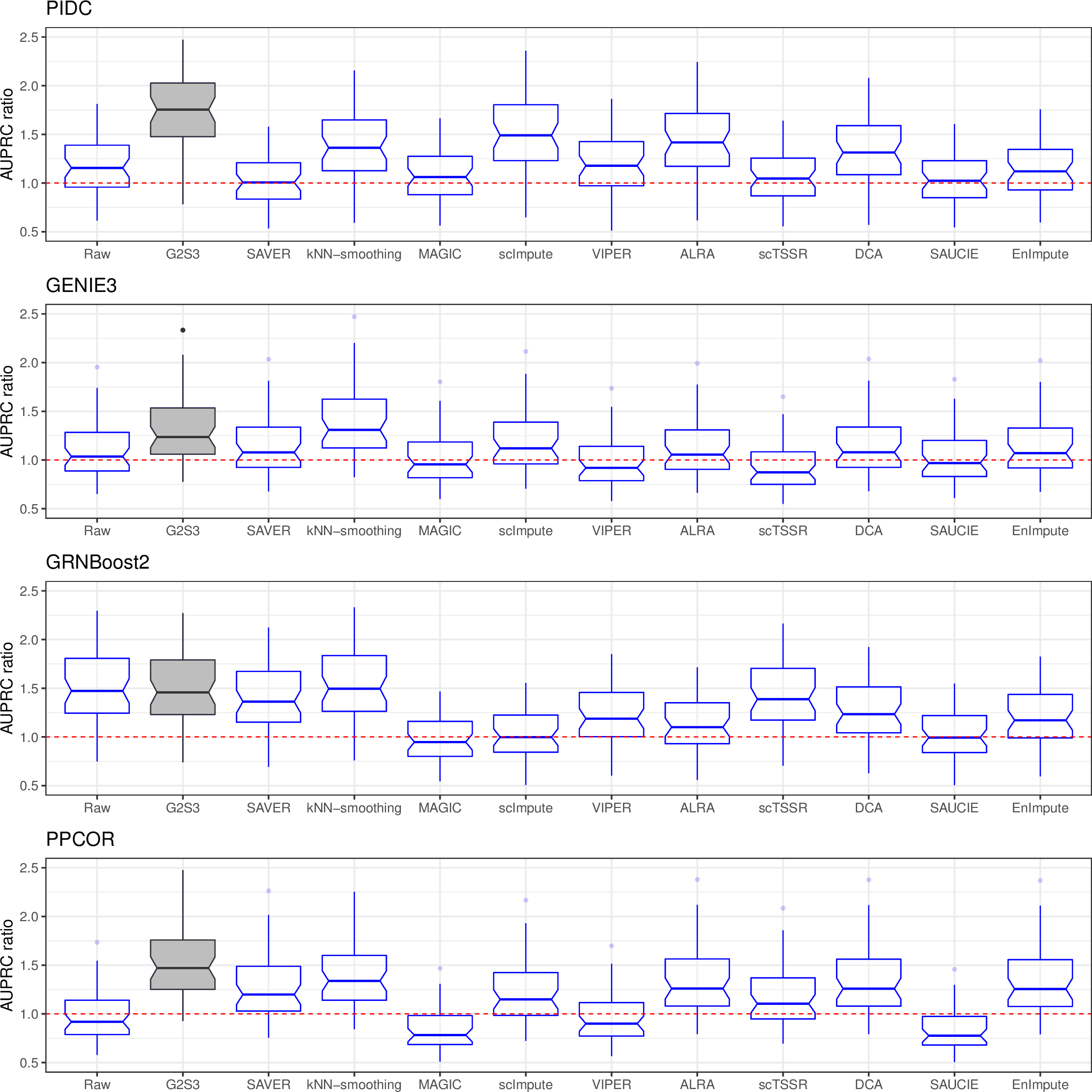

Supplement: S7 Fig — Boxplots showing the area under the precision-recall curve (AUPRC) ratios that measure the accuracy of inferred GRNs using the imputed data by different imputation methods. PIDC, GENIE3, GRNBoost2 and PPCOR are used to infer GRNs. Red line indicates the performance of a random predictor. (TIF) [file pcbi.1009029.s007.tif]

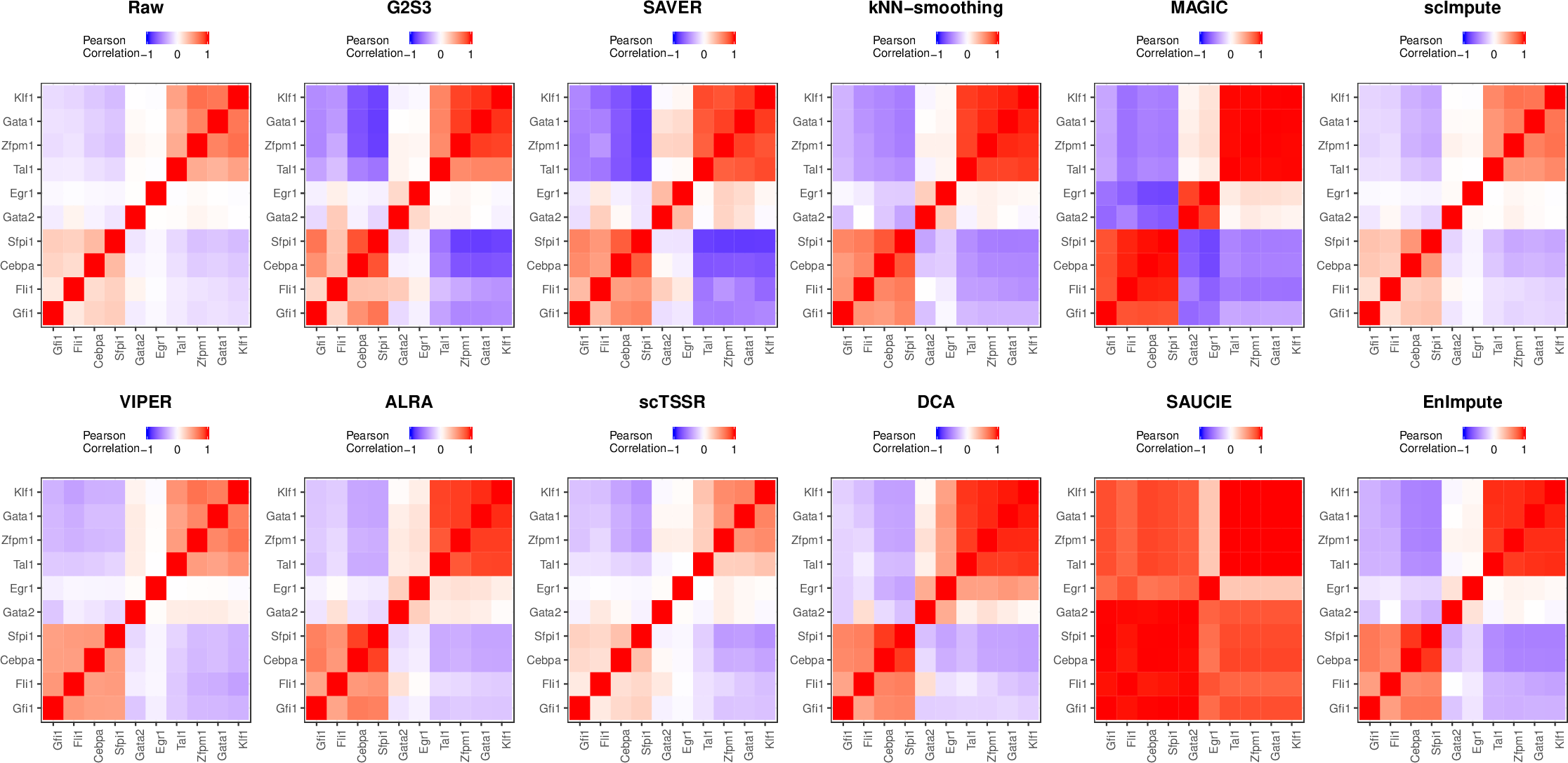

Supplement: S8 Fig — Heatmaps of pairwise correlations between well-known blood regulators. (TIF) [file pcbi.1009029.s008.tif]

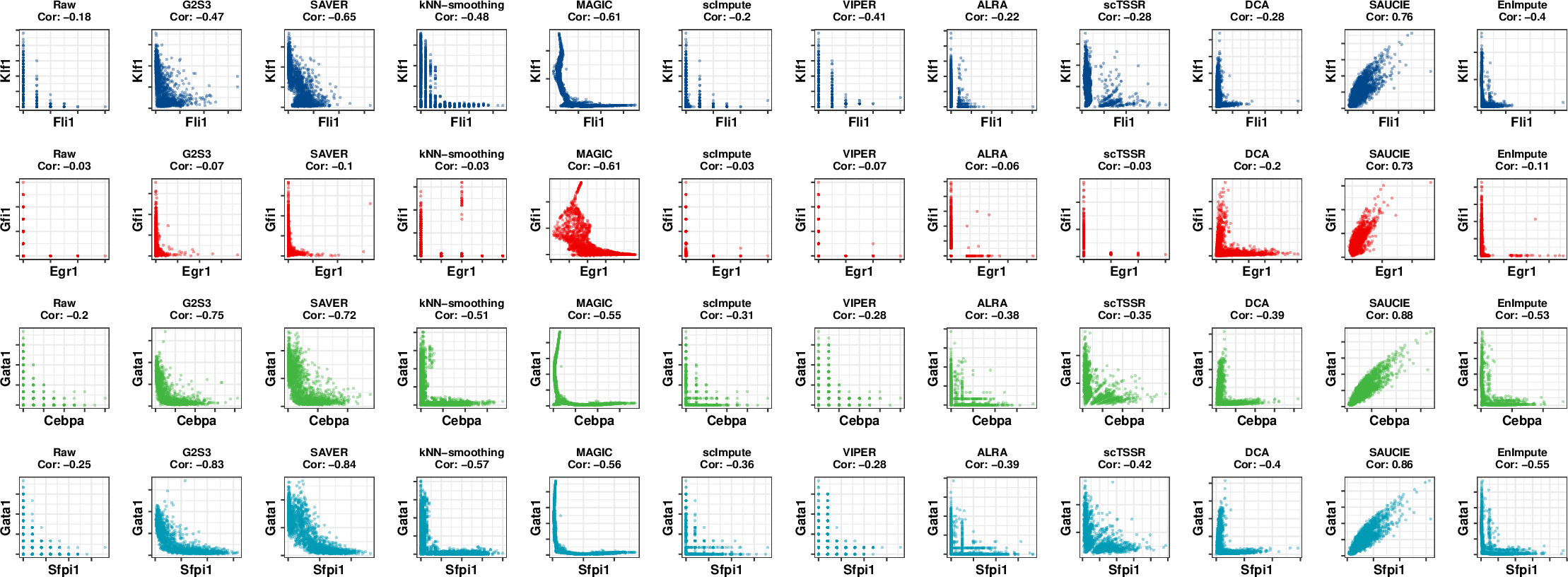

Supplement: S9 Fig — Each row shows the scatterplots of a mutually inhibitory gene pair in the raw and imputed data by all methods. (TIF) [file pcbi.1009029.s009.tif]

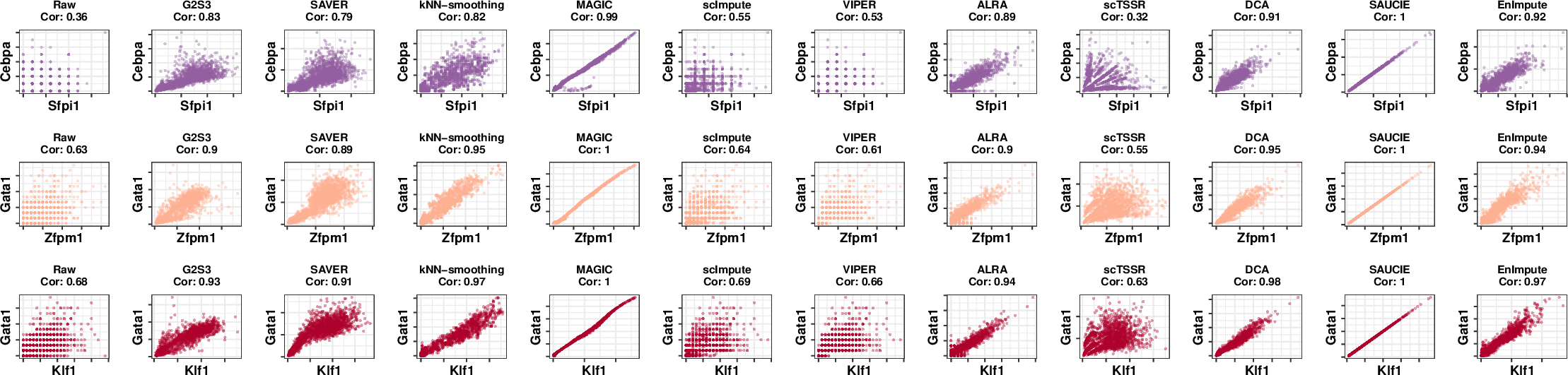

Supplement: S10 Fig — Each row shows the scatterplots of a mutually activatory gene pair in the raw and imputed data by all methods. (TIF) [file pcbi.1009029.s010.tif]
