# Supplementary material for: G2S3: A gene graph-based imputation method for single-cell RNA sequencing data
Source: PLoS Comput Biol. 2021 May 18;17(5):e1009029. doi: 10.1371/journal.pcbi.1009029 (PMC8189489; doi:10.1371/journal.pcbi.1009029)
Supplement: S1 Table — P-values of testing the difference of correlations of G2S3 and other methods with the reference data. (DOCX) [file pcbi.1009029.s011.docx]

**Table S1. Comparison of the gene-wise and cell-wise correlations of G2S3 and other methods in down-sampling experiments.** P-values of testing the difference of correlations of G2S3 and other methods with the reference data.

| **Gene-wise Correlation** | | | | | | | | | | |
| --- | --- | --- | --- | --- | --- | --- | --- | --- | --- | --- |
| **Datasets** | **SAVER** | **kNN-smoothing** | **MAGIC** | **scImpute** | **VIPER** | **ALRA** | **scTSSR** | **DCA** | **SAUCIE** | **EnImpute** |
| Reyfman | < 2.2×10^-16^ | < 2.2×10^-16^ | < 2.2×10^-16^ | < 2.2×10^-16^ | 2.3×10^-10^ | < 2.2×10^-16^ | < 2.2×10^-16^ | < 2.2×10^-16^ | < 2.2×10^-16^ | < 2.2×10^-16^ |
| PBMC | < 2.2×10^-16^ | < 2.2×10^-16^ | < 2.2×10^-16^ | < 2.2×10^-16^ | 6.3×10^-4^ | < 2.2×10^-16^ | 1.4×10^-4^ | < 2.2×10^-16^ | < 2.2×10^-16^ | < 2.2×10^-16^ |
| Zeisel | < 2.2×10^-16^ | < 2.2×10^-16^ | < 2.2×10^-16^ | < 2.2×10^-16^ | < 2.2×10^-16^ | < 2.2×10^-16^ | 8.1×10^-3^ | < 2.2×10^-16^ | < 2.2×10^-16^ | < 2.2×10^-16^ |
| **Cell-wise Correlation** | | | | | | | | | | |
| **Datasets** | **SAVER** | **kNN-smoothing** | **MAGIC** | **scImpute** | **VIPER** | **ALRA** | **scTSSR** | **DCA** | **SAUCIE** | **EnImpute** |
| Reyfman | < 2.2×10^-16^ | < 2.2×10^-16^ | < 2.2×10^-16^ | < 2.2×10^-16^ | < 2.2×10^-16^ | < 2.2×10^-16^ | < 2.2×10^-16^ | < 2.2×10^-16^ | < 2.2×10^-16^ | 1.5×10^-6^ |
| PBMC | 1.7×10^-10^ | < 2.2×10^-16^ | < 2.2×10^-16^ | < 2.2×10^-16^ | < 2.2×10^-16^ | < 2.2×10^-16^ | < 2.2×10^-16^ | < 2.2×10^-16^ | < 2.2×10^-16^ | 2.4×10^-2^ |
| Zeisel | 6.1×10^-9^ | < 2.2×10^-16^ | < 2.2×10^-16^ | < 2.2×10^-16^ | < 2.2×10^-16^ | < 2.2×10^-16^ | < 2.2×10^-16^ | < 2.2×10^-16^ | < 2.2×10^-16^ | < 2.2×10^-16^ |
