# Supplementary material for: G2S3: A gene graph-based imputation method for single-cell RNA sequencing data
Source: PLoS Comput Biol. 2021 May 18;17(5):e1009029. doi: 10.1371/journal.pcbi.1009029 (PMC8189489; doi:10.1371/journal.pcbi.1009029)
Supplement: S2 Table — Runtime in minutes for each imputation task using a single processor on an 8-core, 50 GB RAM, Intel Xeon 2.6 GHz CPU machine. (DOCX) [file pcbi.1009029.s012.docx]

**Table S2. Computational time of all imputation methods.** Runtime in minutes for each imputation task using a single processor on an 8-core, 50 GB RAM, Intel Xeon 2.6 GHz CPU machine. *Computation time summing over five methods and ensemble runtime.

|  | **G2S3** | **SAVER** | **kNN-**  **smoothing** | **MAGIC** | **scImpute** | **VIPER** | **ALRA** | **scTSSR** | **DCA** | **SAUCIE** | **EnImpute*** |
| --- | --- | --- | --- | --- | --- | --- | --- | --- | --- | --- | --- |
| Reyfman | 4.27 | 60.12 | 0.25 | 0.35 | 29.46 | 5289.17 | 0.16 | 9.80 | 5.40 | 0.86 | 102.23 |
| Zeisel | 2.99 | 43.26 | 0.18 | 0.24 | 70.67 | 3618.86 | 0.10 | 4.26 | 4.27 | 0.74 | 121.84 |
| PBMC | 1.09 | 25.91 | 0.15 | 0.17 | 17.77 | 524.37 | 0.08 | 3.48 | 2.78 | 0.98 | 50.84 |
