# Supplementary material for: G2S3: A gene graph-based imputation method for single-cell RNA sequencing data
Source: PLoS Comput Biol. 2021 May 18;17(5):e1009029. doi: 10.1371/journal.pcbi.1009029 (PMC8189489; doi:10.1371/journal.pcbi.1009029)
Supplement: Attachment — Submitted filename: Response_G2S3_PCB.docx [file pcbi.1009029.s013.docx]

We thank Associate Editor and referees for their thoughtful and constructive comments. Below we detail how each of the comments has been addressed. The reviewers’ comments are in italics.

**Response to Reviewer 1:**

1. *In order to support their central argument that “G2S3 is superior in recovering true expression levels”, more computational and biological evidences are needed, especially since many imputation methods have already been developed for single-cell data. The G2S3 method/model also needs better justification with computational evidences.*

We made the following changes to provide more computational and biological evidence to demonstrate the advantage of G2S3. First, we added five additional imputation methods for comparison, including kNN-smoothing, ALRA, scTSSR, SAUCIE and EnImpute. In the revised manuscript, G2S3 is compared with ten existing imputation methods. Second, we added the comparison of method performance in reconstructing cell trajectories. Third, we added one more dataset in the performance comparison in identifying differentially expressed genes. Fourth, we added one more dataset in the performance comparison in recovering gene-gene relationship. Lastly, we added the comparison between G2S3 and other methods in terms of computational cost. These changes can be found throughout the revised manuscript but mostly in the sections **Results**, and **Materials and methods**.

2. *“Down-sampling was applied to these datasets to assess the method performance in recovering true expression levels.” Since ground truth gene expression is in general not available for real data, it is acceptable to use the down-sampling approach to create synthetic datasets for assessing method performance. However, it is very important to demonstrate that the down-sampled data really resemble the real data distribution, so that the conclusions on down-sampled data can be generalized to real data. Did the authors filter out any genes or cells in the down-sampling process? Are the gene expression mean-variance relationship and other relationships between key variables preserved in down-sampled data compared to real data. These questions are also critical since all methods except for G2S3 have poor performance in terms of gene correlation on down-sampled data. Even G2S3’s imputed data does not have a significant better performance than observed data, especially if we look at the worst cases.*

We did filter out genes expressed in a small number of cells and cells with a small total number of UMIs to generate the reference data in each real dataset. A detailed description of the filtering criteria in each dataset can be found in the subsection **Real datasets** on pages 22-24. Then, we randomly masked out 80% of UMIs in each reference data to create the down-sampled observed data without any filtering of genes or cells, to which all imputation methods were applied.

To examine the resemblance in distribution of the down-sampled data to the reference data, we calculated the coefficient of variation (CV) of gene expression before and after down-sampling. In all three datasets used in the down-sampling experiment, although the CV of gene expression increased slightly after down-sampling, the correlation between the CV before and after down-sampling was 0.79 or higher, demonstrating that the down-sampled data well preserved the mean-variance relationship in the reference data (Fig S1).

The comparison in Fig 1 showed no benefit in data imputation mainly due to the large proportion of lowly expressed genes. When we stratified genes into widely expressed, mildly expressed, and rarely expressed genes based on the proportion of cells expressing them in the down-sampled data, G2S3, SAVER, scTSSR and EnImpute imputed data all had better gene-wise correlation than the observed data for widely and mildly expressed genes, but not for rarely expressed genes (Fig S3). In fact, the imputed data by all methods showed worse correlation than the observed data for rarely expressed genes in all three datasets. In addition, the percentages of rarely expressed genes were around 80% in both Reyfman and PBMC datasets and about 45% in the Zeisel dataset. Therefore, when we compared the gene-wise correlation using all genes together shown in Fig 1, the results were highly dominated by the results from rarely expressed genes in both Reyfman and PBMC datasets and showed no benefit by any method. In the Zeisel dataset, the domination of rarely expressed genes was much less due to a smaller percentage of rarely expressed genes and the comparison showed a better gene-wise correlation than the observed data for G2S3 and scTSSR only. The reason for a worse performance of all imputation methods for rarely expressed genes was that these genes do not have enough cells with non-zero nUMI for any imputation methods to accurately detect similar genes to borrow information from for imputation. In the **Discussion** section, we added discussion on this issue and possible future work to improve this.

3. *For the restoration of cell subtype separation, the authors only compared methods based on 1-10 PCs, which only represent a narrow range. What if the authors look at the results with 15, 20, …, 50 PCs?*

We updated Fig 2 to compare methods based on the top 1, 2, …, 50 PCs. In the updated figure, G2S3 and EnImpute had the highest inter/intra-subtype distance ratio and silhouette coefficient. Both methods performed better than the raw unimputed data, while MAGIC, scImpute, ALRA and DCA performed worse than the raw data. SAUCIE performed the worst. These results suggest that G2S3 greatly improved the separation between different cell types by enhancing the biologically meaningful information in the top PCs. Its performance is comparable to EnImpute that takes advantage over several methods.

4. *Figure 2 compares imputed data based on average inter/intra-subtype distance ratio (top) and silhouette coefficient. Since the true cell types are known, the author should also compare the performance based on cell type clustering/classification. Figure 3 demonstrates the PCA plots for the Chu dataset. It would be helpful to also show the tSNE plots. Another question is whether the plots are based on counts or log-transformed counts?*

We calculated these two measures exactly as suggested by this comment. The true cell types were treated as classification of cells. The inter-/intra- cell types distance and silhouette coefficient were calculated to measure how separated these known cell types are in the imputed data by each method. We updated Fig 3 to use UMAP plots which used log-transformed counts.

5. *For the Petropoulos dataset, the authors need to better justify the superior performance of G2S3. First, even though cells from different stages are better separated in G2S3’s imputed data, cells from the same stage are also clustered into several subgroups. The authors need to show that these subgroups are biologically meaningful and are not artifacts of G2S3’s imputation. Second, even though G2S3 and MAGIC both show better results in the previous study, we don’t know if this holds in additional analyses, so the other methods should also be compared on this dataset.*

We added cell trajectory analysis in the Petropoulos dataset to demonstrate the biological meaning of the imputed data (Fig 4). The cell trajectory plots did show the sequential layout of cells from earlier to later embryonic days. The pseudo-time inferred from the G2S3 imputed data showed a high correlation (pseudotemporal ordering score and Kendall rank correlation coefficient) with the actual embryonic days, indicating that the separation between cells in the imputed data by G2S3 was biologically meaningful. Furthermore, the trajectory analysis showed an increased heterogeneity among cells from later embryonic days, especially starting from embryonic day 5. This was consistent with the observation of a significant embryonic cell differentiation event on embryonic day 5 [1]. In addition, we added all other imputation methods for comparison.

6. *For the study on gene regulatory relationship recovery, the authors argue that “G2S3 showed the greatest enhancement of the regulatory relationship…” However, G2S3 and DCA have very similar performance in Figure 6A. It’s also not clear why “MAGIC performed worse than the raw data”. In additional to plotting the correlation matrices in Figure 6, the authors should also compare the methods in a stricter manner – using a quantitative criterion. This study is based a few number of gene pairs. The authors should consider using more gene pairs (e.g., known TF-target pairs) for benchmarking.*

We clarified our description of this result in lines 305-325 on pages 14-15. To compare the methods in a stricter manner, we added the comparison of the correlation of each gene pair to quantify the gene regulatory relationship in the imputed data by all methods in Fig S5 and S6. We also added another scRNA-seq dataset (the Buettner dataset) to compare method performance in recovering gene correlation relationship. This dataset provides scRNA-seq data of cells from different cell cycle stages, which included 2,211 gene pairs to assess the recovery of gene-gene relationship. Since this dataset was used to assess gene correlation relationship instead of regulatory relationship, we changed the title of this section to "Gene correlation relationship recovery".

7. *G2S3 learns a weighted adjacency matrix for genes using an objective function adapted from Kalofolias’s model. Since several methods have been developed for single cell data to construct gene networks, what if the authors use any of these methods to learn the adjacency matrix? What’s the additional advantage of model (1)? Since this is the novel part in G2S3’s imputation framework, it is important to demonstrate why model (1) is the best strategy for learn gene-gene relationships.*

G2S3 learns gene-gene relationship assuming smoothness and sparsity on the gene graph, so the additional advantage of model (1) is that it generates a sparse gene graph and at the same time allows expression levels to change smoothly between closely connected genes. Since many gene networks and biochemical networks are sparse [2–4], the sparsity property is important for inferring gene network. We agree that several methods are available for constructing gene networks, many of them use Gaussian kernel as weights to obtain a smooth network. This type of methods results in full matrices and sparsity is imposed afterwards, for example, thresholding the adjacency weights. We found that top eigenvectors of graph Laplacian on the gene networks learned from Gaussian kernel were highly correlated with dropout rate, suggesting that dropout events tend to bias the construction of gene network in scRNA-seq data. We added discussion on this advantage in the **Discussion** section.

8. *For the imputation step, why is it best to take one step of the random walk? Like for other diffusion-based methods such as MAGIC, the authors need to justify the selection of this key parameter.*

We took one step of the random walk to avoid over-smoothing. Multiple steps of the random walk tended to overly smooth data and lead to worse performance. Similar observations were reported in another manuscript discussing parameter tuning for diffusion-based imputation methods for scRNA-seq data [5]. Their results showed that for many diffusion-based methods including MAGIC, single step (t=1) yielded better performance than multiple steps or iterations until convergence. We added discussion on this in the **Discussion** section.

9. *Is any normalization and data transformation methods applied on the count matrix X before applying G2S3?*

G2S3 does not require normalization or data transformation because it allows raw count as an input. It can also be applied to a normalized or transformed dataset. In this article, no normalization was performed before imputation by G2S3. G2S3 is robust to variations in sequencing depth across cells that it performed well in both UMI-based and RPKM datasets used in our experiments. We performed normalization and transformation for performance evaluation when comparing different methods. The description can be found in the subsection **Performance evaluation** on page 24.

**Response to Reviewer 2:**

1. *As the authors cited and stated, the key model (Equation 1) was adapted from a previous Kalofolias’s model. The novelty of the proposed method is unclear. In addition, there are three terms in the proposed objective function (Equation 1). In most machine learning studies, two tuning parameters are often added to balance the contribution of each term. It would be better if the authors can add two other tuning parameters and analyze the effect of each term on the results.*

Although part of our model was adapted from a previous Kalofolias’s model, our method is still novel in the sense that it is the first to use graph signal processing technique for scRNA-seq data imputation. We fully acknowledge the limitation of our current model in which we set both tuning parameters to be 1. In the **Discussion** section, we added discussion on future work to tune the parameters based on the complexity and structure of scRNA-seq datasets.

2. *Now there are a lot of methods that have been proposed for imputing dropout events in scRNA-seq data in the last three years (*[*https://www.scrna-tools.org/tools?sort=name&cats=Imputation*](https://www.scrna-tools.org/tools?sort=name&cats=Imputation)*). The authors just compared their method with five existing methods. More methods developed recently (e.g., ALRA, EnImpute, kNN-smoothing, scTSSR, SAUCIE) should be compared.*

We added these five methods for comparison in the revised manuscript.

3. *The authors did not consider SAVER in some experiments since, as they explained, it was not able to finish imputation within 24 hours for large scale datasets. In order to compare the proposed method with SAVER, the authors can run the imputation methods on datasets with only selected genes (e.g., highly variable genes).*

We added SAVER results to all the experiments in the revised manuscript.

4. *In the differential expression analysis and gene regulatory relationship analysis experiments, only one dataset is used. In order to evaluate the generalization of the proposed methods, more datasets should be considered in these two experiments.*

We added the Chu dataset to compare method performance in identifying differentially expressed genes, results can be found in lines 271-283 on pages 13-14 and Fig 5. We also added the Buettner dataset to compare method performance in recovering gene correlation relationship, results can be found in lines 331-343 on pages 15-16 and Table 2.

5. *It would be better if the authors can test the performance of different methods on other downstream tasks of scRNA-seq analysis (e.g., cell trajectory inference).*

We added the method comparison based on cell trajectory analysis using the Petropoulos dataset, which measured expression profiles of embryonic cells from different embryonic days (Fig 4). The inferred trajectory was visualized for comparison. In addition, the actual embryonic days of cells were considered as the ground truth in the cell trajectory analysis and we evaluate method performance based on the consistency between the inferred pseudo-time from trajectory analysis and the actual embryonic days of cells. The pseudotemporal ordering score and Kendall rank correlation coefficient were calculated to measure the consistency for comparison. Description of the results can be found in the subsection **Improvement in cell trajectory inference** on page 11.

6. *Fig 1: from my viewpoint, the correlation on the gene level after imputation is too low (~0.5), even just slightly higher than ‘observed’ group. I have two concerns: (1) It was meant to impute dropout (i.e., zeroes), but now it seems that all the expression values were locally averaged. (2) Is it necessary to make a slight improvement on correlation but change the whole expression profile? It’s hard to tell whether an imputation method performs well, just relying on the evaluation of gene expression correlations. Moreover, Fig 1, left panel: G2S3 median is lower than that of the observed group on Reyfman and PBMC datasets, and the variation in G2S3 is higher than observed for all datasets (also for the right panel, cell level correlation). So I don’t agree with the following statement: “In all datasets, G2S3 consistently achieved the highest correlation with the reference data at both gene and cell levels”. The superior of G2S3 was not demonstrated.*

The low gene-wise correlation after imputation in Fig 1 was mainly due to the large proportion of lowly expressed genes. When we stratified genes into widely expressed, mildly expressed, and rarely expressed genes based on the proportion of cells expressing them in the down-sampled data, G2S3, SAVER, scTSSR and EnImpute imputed data all had better gene-wise correlation than the observed data for widely and mildly expressed genes, but not for rarely expressed genes (Fig S3). In fact, the imputed data by all methods showed worse correlation than the observed data for rarely expressed genes in all three datasets. In addition, the percentages of rarely expressed genes were around 80% in both Reyfman and PBMC datasets and about 45% in the Zeisel dataset. Therefore, when we compared the gene-wise correlation using all genes together shown in Fig 1, the results were highly dominated by the results from rarely expressed genes in both Reyfman and PBMC datasets and showed no benefit by any method. In the Zeisel dataset, the domination of rarely expressed genes was much less due to a smaller percentage of rarely expressed genes and the comparison showed a better gene-wise correlation than the observed data for G2S3 and scTSSR only.

The reason for a worse gene-wise correlation after imputation than the observed data for rarely expressed genes was that these genes do not have enough cells with non-zero nUMI for any imputation methods to accurately detect similar genes to borrow information from for imputation. In the **Discussion** section, we added discussion on this issue and possible future work to improve this.

Across all three datasets and across widely, mildly and rarely expressed genes, G2S3 had better performance than other methods except scTSSR, which had comparable performance to G2S3. Moreover, the advantage of G2S3 over other methods increases when gene expression level decreases.

7. *Fig 3: Why did the authors use PCA but not tSNE or other dimensional reduction methods for scRNA-seq data?*

We updated Fig 3 to UMAP plots for visualization.

8. *Fig 4: the dataset of human embryo stem cells from different embryonic days was used to analyze cell types. But it should be more appropriate to analyze cell differentiation trajectory here. See also the above comment 5.*

We added the method comparison based on cell trajectory analysis using the Petropoulos dataset (Fig 4). The inferred trajectory was visualized for comparison. In addition, the actual embryonic days of cells were considered as the ground truth in the cell trajectory analysis and we evaluate method performance based on the consistency between the inferred pseudo-time from trajectory analysis and the actual embryonic days of cells. The pseudotemporal ordering score and Kendall rank correlation coefficient were calculated to measure the consistency for comparison. Description of the results can be found in the subsection **Improvement in cell trajectory inference** on page 11.

9. *Fig 5: The authors divided the dataset into high-expressed genes and low-expressed genes. But in practice, one would not perform such a step for differential expression (DE) analysis. How about the overall performance of different methods on the whole dataset? For bulk expression data, the authors used T test for DE analysis. Why not using the standard pipeline of DE analysis, such as DESeq2?*

We divided genes into highly and lowly expressed genes to demonstrate the impact of dropout rate on the imputation method performance. As we know, lowly expressed genes have higher dropout rate and thus have less number of cells with accurate expression levels for any imputation methods to accurately identify similar genes to borrow information from for imputation. The high dropout rate of these lowly expressed genes present a harder task for imputation methods to recovery for DE analysis as they often leads to high false positive rates, as reported in previous studies [6,7]. The DE results on the whole dataset showed that MAGIC had the largest AUC of 0.88. All other imputation methods performed similar to that of the raw data (Fig A below). The high power of MAGIC was driven by its high AUC in highly expressed genes which take up more than 80% of the genes in the dataset.

Figure A: ROC curve for overall differential expression analysis based on DE genes identified by t-test in bulk RNA-seq data in the Segerstolpe dataset

We did not use DESeq2 because the bulk expression data in the Segerstolpe dataset is RPKM (floating points) while DESeq2 takes read counts and uses a negative binomial model derived test for the DE analysis. Regardless we still applied DESeq2 to the bulk expression data by rounding floating-point numbers up to integers. The DE results on the whole dataset based on the ground truth identified by DESeq2 in bulk RNA-seq data showed that G2S3 and MAGIC achieved comparably high AUC. All other methods did not have power to detect DE genes in the dataset (Fig B below).

Figure B: ROC curve for overall differential expression analysis based on DE genes identified by DESeq2 in bulk RNA-seq data in the Segerstolpe dataset

10. *Fig 6: it’s hard to tell which method is better for GRN recovering using correlation scatter plot. The results of this part are more descriptive but not quite rigorous.*

We added the comparison of the correlation of each gene pair to quantify the gene regulatory relationship in the imputed data by all methods in Fig S5 and S6 for a more rigorous comparison. We also added another scRNA-seq dataset (the Buettner dataset) to compare method performance in recovering gene correlation relationship. This dataset provides scRNA-seq data of cells from different cell cycle stages, which included 2,211 gene pairs to assess the recovery of gene-gene relationship. Since this dataset was used to assess gene correlation relationship instead of regulatory relationship, we changed the title of this section to "Improvement in restoring gene correlation relationships". This result can be found in lines 331-343 on pages 15-16.

11. *Please use a table to list computation times of all the compared methods for all the analyzed datasets.*

We added Table S1 to tabulate the computation time of all methods.

12. *Page 17, please check whether the following text are typos: Line 353: “2tr(…)”? Line 354: “L”?*

Eq. (1) is equivalent to $2 \mathrm{tr}\left( X^{T}LX \right)$ where $L$ is the graph Laplacian and $\mathrm{tr}\left( . \right)$ is the trace of a matrix. We corrected this in line 449 on page 21.

**Reference:**

1.	Petropoulos S, Edsgärd D, Reinius B, Deng Q, Panula SP, Codeluppi S, et al. Single-Cell RNA-Seq Reveals Lineage and X Chromosome Dynamics in Human Preimplantation Embryos. Cell. 2016 May;165(4):1012–1026.

2.	Cai X, Bazerque JA, Giannakis GB. Inference of Gene Regulatory Networks with Sparse Structural Equation Models Exploiting Genetic Perturbations. PLOS Computational Biology. 2013 May 23;9(5):e1003068.

3.	Qiu X, Mao Q, Tang Y, Wang L, Chawla R, Pliner HA, et al. Reversed graph embedding resolves complex single-cell trajectories. Nature Methods. 2017 Oct;14(10):979–82.

4.	Kikkawa A. Random Matrix Analysis for Gene Interaction Networks in Cancer Cells. Scientific Reports. 2018 Jul 13;8(1):10607.

5.	Optimal tuning of weighted kNN- and diffusion-based methods for denoising single cell genomics data | bioRxiv [Internet]. [cited 2020 Oct 7]. Available from: https://www.biorxiv.org/content/10.1101/2020.02.28.970202v1.abstract

6.	Andrews TS, Hemberg M. False signals induced by single-cell imputation. F1000Res [Internet]. 2019 Mar 5 [cited 2020 Apr 7];7. Available from: https://www.ncbi.nlm.nih.gov/pmc/articles/PMC6415334/

7.	Soneson C, Robinson MD. Bias, robustness and scalability in single-cell differential expression analysis. Nature Methods. 2018 Apr;15(4):255–61.
