# Supplementary material for: G2S3: A gene graph-based imputation method for single-cell RNA sequencing data
Source: PLoS Comput Biol. 2021 May 18;17(5):e1009029. doi: 10.1371/journal.pcbi.1009029 (PMC8189489; doi:10.1371/journal.pcbi.1009029)
Supplement: Attachment — Submitted filename: Response_G2S3_PCB_rev2_final.docx [file pcbi.1009029.s014.docx]

We thank Editor, Associate Editor and referees for the time reading our paper and providing us thoughtful and constructive comments. The main concern in the second-round review was that the performance advantage of our method in comparison to many other existing methods did not appear to be clear and convincing. We apologize for not clearly presenting the overall performance of our method in comparison to others. We have carefully revised our manuscript to clearly demonstrate the advantage of our method. Below we detail how each reviewer’s comment has been addressed. We appreciate if our manuscript can be reconsidered for publication. The reviewers’ comments are in italics.

**Response to Reviewer 1:**

1. *The authors did not include line numbers of the revised contents in the response letter, nor did they highlight/color the revised parts in the updated manuscript, making it difficult to evaluate if some of my previous comments (in particular, comments 1, 6, and 8) are fully addressed.*

We apologize for this inconvenience. It is unclear to us how this happened. In the last round of revision, we did submit both a marked version and a clean version of our revised manuscript. We also included the section information and page/line numbers to highlight changes in response to reviewer’s comments. For this revision, we have included the line numbers of the revised contents in this response letter and highlighted all revisions in the submitted manuscript.

2. *For the procedure to filter out genes, the authors applied different sets of thresholds for each dataset. What is the rationale for selecting these thresholds? Some of the thresholds seem to be very stringent and would not be used in real practice. For example, in the Segerstolpe data, the authors used “filtering to remove genes expressed in less than 20% of cells”.*

We used similar cutoffs as those in the down-sampling experiments in the SAVER paper. These stringent cutoffs selected genes expressed in a large proportion of cells so that the reference datasets were more reliable, i.e., the filtering criteria on genes were 20%-40%. In the Buettner dataset used in gene correlation analysis, we aimed to assess the correlation of 67 periodic marker genes and used a filtering threshold of 20% to obtain a subset of 13,355 genes. The netNMF-sc paper analyzed the same dataset with a more stringent cutoff for filtering to obtain a smaller subset of 9,571 genes. In the other datasets demonstrated in our manuscript, we used either 10% or less stringent cutoffs.

3. *I still have one concern about Figure 2. Why don’t the authors calculate and report the adjusted rand index or normalized mutual information between inferred clusters and true cell types, both of which are commonly used in single-cell studies to evaluate clustering performance?*

We have added the normalized mutual information (MI) and adjusted rand index (RI) to measure the consistency between cell clustering results and true cell subtype labels in Fig 3. Based on these two measures, EnImpute had the best separation of the cell subtypes (MI=0.77, RI=0.70) and G2S3 was the second best (MI=0.74, RI=0.64), while the other methods did not have comparable performance. We have also added MI and RI in S5 Fig which showed the best performance of G2S3, without any other method having comparable performance. Notice that EnImpute is an ensemble method that combines multiple imputation results, and G2S3 is the only method that achieved comparable performance to EnImpute. These changes can be found in Fig 3, S5 Fig, lines 205-207, 219-224, 233-235, 237-241 on pages 10-12 in the subsection **Restoration of cell subtype separation**.

4. *“To avoid over-smoothing” is not a solid justification for taking one step of the random walk. I agree that too many steps may result in over-smoothing in imputation, but there should be a more model-guided way to determine the optimal number of steps instead of arbitrarily choosing one.*

We agree that a model-guided approach to determine the optimal number of steps is needed. We have added an approach to determine the optimal value of steps in our method (lines 512-518 on page 24) as follows: The imputed count matrix $X_{\mathrm{imputed}}$ is obtained by taking a $t$-step random walk on the graph which can be written as $X_{\mathrm{imputed}}^{T}=M^{t}\times X^{T}$. By default, G2S3 takes a one-step random walk ($t=1$) to avoid over-smoothing. We also implement an option of tuning the hyperparameter $t$ based on an objective function that minimizes the mean squared error (MSE) between the imputed and observed data, i.e., $t^{*}=\underset{t}{\mathrm{argmin}} \left\| M^{t}X^{T}-X^{T} \right\|$.

We also evaluated this tuning procedure on the hyperparameter $t$ by comparing the MSE as well as the gene-wise and cell-wise correlations of the G2S3 imputed data with reference data, for $t=1,\ldots,10$, in each of the three down-sampled datasets. The optimal value of $t$ is 1 in all three datasets based on the minimization of MSE. In addition, the one-step random walk in G2S3 achieved the greatest gene-wise and cell-wise correlations with the reference data. These evaluation results were presented in a new subsection **Hyperparameter tuning in G2S3** on pages 7-8.

5. *The authors’ argument that “G2S3 is robust to variations in sequencing depth across cells that it performed well in both UMI-based and RPKM datasets used in our experiments.” is not convincing to me. If G2S3 does not require normalization before imputation, how does it account for the varying sequencing depths in different individual cells? This is necessary for accurate imputation of gene expression levels.*

For UMI-based datasets, variations in sequencing depth can influence the estimation of gene-gene relationships when constructing gene graph in G2S3. Therefore, we recommend normalizing UMI counts before applying G2S3. For RPKM/FPKM datasets, G2S3 can be directly applied. In all the analyses presented in the manuscript, all UMI-based datasets were normalized before G2S3 imputation. We have added this recommendation in lines 458-460 on page 21 in **Discussion**.

**Response to Reviewer 2:**

1. *In the revised manuscript, the authors have compared G2S3 with several other imputation methods. But unfortunately, G2S3 does not show dominant or convincing superiority in most tasks. For example, in correlation with reference, G2S3 is not significantly higher than scTSSR; in cell trajectory inference, G2S3 is not better than scImpute and VIPER; in differential expression analysis, G2S3 is not better than MAGIC. In addition, gene-gene relationship recovery is not convincingly demonstrated.*

In Fig 7 of the revised manuscript, we have summarized the overall performance of G2S3 in comparison with the other ten imputation methods across the five evaluation criteria: recovering true expression levels, restoring cell subtype separation, reconstructing cell trajectory, identifying differentially expressed genes, and restoring gene regulatory and correlation relationship. G2S3 was ranked first in three out of the five evaluation criteria, second in cell clustering, and third in cell trajectory inference. For those criteria under which G2S3 did not achieve the best performance, it had close or comparable performance to the best method. No other method achieved the best performance in as many criteria as G2S3. Overall, G2S3 performed the best among all the methods, followed by EnImpute and VIPER. These changes can be found in Fig 7 and a new subsection **Summary of method performance** on page 18.

Specifically, in expression data recovery, we have added one-sided t-test to compare the gene-wise and cell-wise correlations of G2S3 to those of the other methods. G2S3 achieved significantly higher correlations with the reference data than all the other methods across three datasets for both gene-wise and cell-wise correlations (p<0.05, S1 Table). This demonstrated that G2S3 had a significantly better performance than the other methods in correlation with reference. In cell clustering, G2S3 performed the second best after EnImpute. In cell trajectory inference, G2S3 was ranked third after scImpute and VIPER (Fig 4). In differential expression analysis, we presented the predictive power across all genes in the Chu and Trapnell datasets. G2S3 achieved the highest area under the curve (AUC) in both datasets (Fig 5). In the gene-gene relationship recovery, we added the analysis using the inferred gene regulatory network (GRN) to assess the network prediction accuracy in the Paul dataset. G2S3 achieved the highest area under the precision-recall curve (AUPRC) ratio among all methods (Fig 6).

2. *Fig 1 shows that G2S3 does not perform best, particularly compared to scTSSR which has already considered not only gene similarity but also cell similarity for single cell gene expression imputation. The method novelty and performance improvement of G2S3 is a big problem of my concern. The statistical significance for comparisons was not provided, which should be provided at least in the supplementary information.*

We have quantified the performance advantage of G2S3 in our revised manuscript. One-sided t-test was applied to compare the gene-wise and cell-wise correlations of G2S3 to those of the other methods. G2S3 achieved significantly higher correlations with the reference data than all the other methods, including scTSSR, across three datasets for both gene-wise and cell-wise correlations (p<0.05, S1 Table). We described this result in lines 156-159 on page 8.

3. *Fig 5: I don’t think it’s reasonable to divide the whole gene set into highly-expressed genes and lowly-expressed genes, although the authors argued in the response letter that this procedure was for investigating the impact of dropout on imputation (“We divided genes into highly and lowly expressed genes to demonstrate the impact of dropout rate on the imputation method performance”). In my viewpoint, one can perform other more straightforward analysis to directly demonstrate the impact of dropout on the imputation performance, rather than to look at differential expressions. Actually, most literature of imputation methods rarely divide the gene set into highly-expressed genes and lowly-expressed genes for evaluating and benchmarking their performance in DE analysis. The authors also stated that “The high dropout rate of these lowly expressed genes present a harder task for imputation methods to recovery for DE analysis”. If so, does it mean that the imputation methods would perform better on the highly-expressed genes to recovery for DE analysis? But the results in Fig 5 (both A and B, left panels) showed that almost all the methods performed not that well on the highly-expressed genes and even worse than that on the lowly-expressed genes. How to interpret it? The results in Fig 5 are not convincing for me to believe G2S3 is a better imputation method for DE analysis, especially by looking at the results for overall differential expression analysis (Figure A in the response letter). Although the authors emphasized that G2S3 improved DE analysis for the lowly-expressed genes, but it shows much worse performance for the highly-expressed genes. Even for the lowly-expressed genes as the authors emphasized, how to determine a gene is lowly-expressed is also a problem in practice. In addition, MAGIC is not used for comparison for the lowly expressed genes (Fig 5A, right panels)?*

We agree that stratifying genes into highly-expressed and lowly-expressed genes is not commonly seen when assessing the impact of imputation in differential expression analysis in literature. In the revised manuscript, we presented the prediction power using all genes in the Chu and Trapnell datasets. G2S3 achieved the highest area under the curve (AUC) in both datasets (Fig 5). In the Trapnell dataset, no genes achieved significance in the imputed data by MAGIC, thus MAGIC was not shown in Fig 5B. SAUCIE failed to identify any significant genes in both datasets, thus was not shown. These results were presented in lines 284-300 on page 14.

4. *To evaluate the performance on gene-gene relationship recovery, Table 2 makes some sense but Fig 6 (as well as the related Fig S5-S6) is not very reasonable by examining whether the pair-wise correlation was enhanced since the true correlation is unknow, unless the authors could use, for instance, FISH data for testing (See Fig S3 in Ref [14]). To demonstrate G2S3 could help improve the gene network reconstruction, the authors may consider to perform GRN inference based on the data imputed by G2S3 or other imputation methods and to assess whether the network prediction accuracy (e.g., AUROC or AUPRC) could be increased compared to the raw data. The scRNA-seq data based GRN inference methods as well as the benchmarking dataset can be referred to the following paper: Pratapa, A., Jalihal, A.P., Law, J.N. et al. Benchmarking algorithms for gene regulatory network inference from single-cell transcriptomic data. Nat Methods 17, 147–154 (2020).*

In our revised manuscript, we reconstructed gene regulatory network (GRN) using GENIE3 and PPCOR on the raw and imputed datasets in the Paul dataset to assess the network prediction accuracy. G2S3 achieved the highest area under the precision-recall curve (AUPRC) ratio, AUPRC divided by that of a random predictor with 50 replications, followed by kNN-smoothing, in both GRN inference methods (Fig 6). The AUPRC ratios of GRNs inferred from the imputed data by either MAGIC or SAUCIE were much lower than that from a random predictor, suggesting that the gene regulatory relationships were distorted after imputation. We added these results in lines 317-326 on page 15.

5. *Fig 2: It’s hard to distinguish between different curves. A better presentation should be considered. In addition, color-annotation for different methods in Fig 1, Fig 2 and Fig 5 seem not consistent.*

We have changed the color annotation so that they are consistent and more distinguishable in Figs 1, 2 and 5.
