# Supplementary material for: G2S3: A gene graph-based imputation method for single-cell RNA sequencing data
Source: PLoS Comput Biol. 2021 May 18;17(5):e1009029. doi: 10.1371/journal.pcbi.1009029 (PMC8189489; doi:10.1371/journal.pcbi.1009029)
Supplement: Attachment — Submitted filename: Response_G2S3_PCB_rev3_final.docx [file pcbi.1009029.s015.docx]

We thank Associate Editor and referees for their thoughtful and constructive comments. Below we detail how each of the comment was addressed. The reviewers’ comments are in italics.

**Response to Reviewer 2:**

1. *“G2S3 algorithm” in the Methods section should be more carefully and clearly described.
   For example, “1” in the second term of equation (1) was not explained and it seems that this symbol was not standardly typed—it’s hard to be differentiated from the number 1.
   Also, 1{≥0} in equation (2) was not clear. Please refer to ref. 44 for mathematical symbols.
   Moreover, “×” in equation (4) and the following “ ” (Line 518) are not consistent. Check it also in Algorithm 1.*

We have modified the notations to make them clearer. “$1$” in the second term of Equation (1) represents a vector of ones. In the revised manuscript, we changed it to bold font “$\boldsymbol{1}$” to differentiate it from the number 1. $1_{\left\{ w\geq0 \right\}}$ in Equation (2) represents the indicator function that takes value 0 when the condition in the brackets is satisfied, infinite otherwise. We changed the symbol to $\mathbb{I}_{\left\{ w\geq0 \right\}}$. We also removed “×” in Equation (4) to be consistent with the equation in line 538. All notations in Algorithm 1 have been modified to be consistent with what presented in the main text. All these changes can be found in lines 513, 524-526 on pages 23-24.

2. *Assumptions underlying the objective function of MSE should be described to explain its rationality used for selecting optimal step parameter.*

We assume that a good imputation method is not expected to deviate too far away from the raw data structure in the process of denoising. The rationality of using the objective function that minimizes the MSE between the imputed and observed data to select optimal step parameter is to denoise the observed gene expression through attenuating noise due to technical variation while preserving biological structure and variation. The same objective function was also used in a previous study on diffusion-based imputation method (ref. 31). We added this explanation in lines 539-542 on page 25.

3. *I would suggest to move “Hyperparameter tuning in G2S3” in the Results section to the Methods section (after “G2S3 algorithm”), to make it more coherent. Also, Fig S2 could be moved to the main text, as it would strengthen the method part of this manuscript.*

We appreciate the suggestion. We used the MSE criterion for hyperparameter tuning as a heuristic approach following a recent study (ref. 31). Although we performed a validation experiment for this tuning procedure using real datasets, there is no theoretical proof of its optimality. It is possible that other criterion may give better hyperparameter tuning, but proving or finding it is beyond the scope of this study. Therefore, we consider it more appropriate to keep this subsection as auxiliary materials in Results and Fig S2 in supplement. However, we added clarification and reference on hyperparameter tuning in Discussion in lines 482-486 on page 22.

4. *In the previous versions of the manuscript (including R1 version), Segerstolpe dataset was used for benchmarking. But in this revised version (R2), this dataset was changed to Trapnell dataset. Why? It seems that the authors only artificially selected results that are favorable for G2S3.*

We decided not to use the Segerstolpe dataset for benchmarking using the criterion based on differential expression analysis due to the following reason. The bulk RNA-seq in the Segerstolpe dataset measured the average expression profile of cells in human pancreatic islets, which consist of at least five distinct endocrine cell types including $\alpha$ cells, $\beta$ cells, $\gamma$/pp cells, $\delta$ cells, and $\epsilon$ cells. These cell types have distinct expression profiles, so the differentially expressed genes identified from the bulk RNA-seq data are not cell type specific and strongly impacted by cell-type composition changes. Therefore, findings from the scRNA-seq data do not and are not supposed to agree well with those from the bulk RNA-seq data in the Segerstolpe dataset. This makes the Segerstolpe dataset inappropriate for performance evaluation based on the consistency between differentially expressed genes in the bulk RNA-seq and scRNA-seq data. In addition, the Segerstolpe dataset had never been used for performance comparison in any published scRNA-seq data imputation paper.

We realized this critical issue of the Segerstolpe dataset during round 2 revision (R2) and searched for a valid dataset that had been used for method comparison using the same criterion in published scRNA-seq data imputation papers. The Trapnell dataset was found and thus used to replace the Segerstolpe dataset for this evaluation criterion. The bulk RNA-seq data in both the Chu and Trapnell datasets measured the average expression profile of cells from the same cell type, so the differentially expressed genes identified from the bulk RNA-seq data were cell type specific. The Chu dataset measured 19 bulk samples, in which each sample consisted of cells from only one of the seven cell types. The Trapnell dataset measured only one cell type, human skeletal muscle myoblasts, with and without differentiation induction. Therefore, the differential analysis results in the bulk RNA-seq and scRNA-seq data were expected to be consistent in each of the two datasets. In addition, both the Chu and Trapnell dataset had been used for performance comparison using the same evaluation criterion in published scRNA-seq data imputation papers, including scImpute, VIPER, scTSSR, AutoImput and McImpute. To clarify the dataset selection process, we added descriptions on the appropriate dataset setting for this evaluation criterion in lines 278-284 on pages 13-14.

5. *For the Chu dataset, in the previous versions (R1), the DEGs were analyzed by comparing DE and H1 cells. However, in the current version (R2), the DEGs were for NP vs H1 cells. This is also reflected in Line 547 (Methods section). I have the same concern with the above comment that the authors only artificially selected results that are favorable for G2S3. To address the above issues, the authors should perform a more justified evaluation and comparison of DEG analysis. I would like to see new results on more comprehensive datasets and cell types as mentioned above.*

In the previous version (R1), we selected H1 and DE cells for comparison based on what were used in the VIPER paper. During revision R2, we noticed that DE cells had two or more sub-clusters in UMAP and one subcluster was mixed with EC cells (Fig 3). The heterogeneity of DE cells was also noted in the original paper stating “…DE cells showed the greatest heterogeneity…” (ref. 26). Because subclusters affect the consistency of differentially expressed genes between H1 and DE cells, we decided to use a more homogeneous cell type, i.e., NP cells, and compared H1 vs. NP cells. In this revision, we compared H1 cells with all other cell types except H9, which is the same cell type as H1 (human ES cells), to demonstrate results on comprehensive cell types. The results on three out of the four cell types compared to H1 cells demonstrated the best performance of G2S3. TB cells is the only cell type for which G2S3 did not achieve the best performance. All other methods, except EnImpute, did not achieve higher AUC than the raw data, indicating the lack of benefit by performing data imputation for genes differentially expressed between H1 and TB cells, regardless of imputation methods. This result was added in lines 294-302 on page 14 and Fig S6.

6. *How ROCs were generated is not clear. Did the authors use fold changes of genes in scRNA-seq as score to be compared with “ground truth” (i.e., top 200 DEGs in bulk RNA-seq), for calculating ROC?*

We used P-values of genes in scRNA-seq as score to be compared with ground truth for calculating ROC. We added clarification as follows: “The prediction accuracy was measured by the area under an ROC curve by comparing the differentially expressed genes identified in the raw and imputed scRNA-seq data at different P-value threshold with ground truth” in lines 667-670 on page 31.

7. *The authors used Paul dataset to infer GRN. The ground truth of the GRN was not listed out so it’s not clear how they evaluated the network prediction accuracy. The Ref 29 only provides gene targets of 4 TFs (in its supplementary materials). How did the authors use this information as ground truth? How many genes in the Paul dataset were used to reconstruct GRN? How they evaluated the prediction accuracy? Details are needed.*

We used a Boolean network constructed by a systematic review on the interactions of core transcription factors to model myeloid differentiation (ref. 35) as ground truth. The same network was used in the DCA paper. Among the eleven key regulators in the network with known inhibitory and activatory relationships in blood development, ten were present in the Paul dataset. We reconstructed GRN on these ten regulators in the raw and imputed datasets by different methods, using PIDC, GENIE3, GRNBoost2 and PPCOR. The prediction accuracy of each method was evaluated by comparing the inferred GRN to the ground-truth network using AUROC and AUPRC. The AUROC/AUPRC ratio was calculated by dividing AUROC/AUPRC by that of a random predictor and the process was repeated for 50 times. Details have been added in lines 674-684 on page 31.

8. *The author only used GENIE3 and PPCOR for evaluation. However, there are many other methods for network reconstruction. At least, PIDC, GENIE3 and GRNBoost2 should be included, which have been shown as top performing method (Nat Methods 17, 147–154 (2020).* [*https://doi.org/10.1038/s41592-019-0690-6*](https://doi.org/10.1038/s41592-019-0690-6)*). The addition of these methods for evaluation would be beneficial or the readers to assess which GRN method could be used in combination with G2S3 imputation.*

The extensive assessment of imputation methods in GRN inference exceeds the scope of this paper. After all, the focus of our paper is not a systematic review of the performance of imputation methods in GRN inference, but a comparison of different imputation methods. Nevertheless, to accommodate the reviewer’s request, we added two additional methods, PIDC and GRNBoost2, for evaluation. Fig 6 shows that G2S3 achieved the highest AUROC ratio in three out of the four GRN inference methods and performed slightly worse than scImpute using GENIE3. The prediction accuracy of scImpute was much lower than a random predictor using GRNBoost2 and PPCOR. The AUROC ratios of GRNs inferred from the imputed data by MAGIC and ALRA were either equal to or much lower than that from a random predictor, suggesting that the gene regulatory relationships were distorted after imputation. S6 Fig demonstrates the results based on the AUPRC ratio. G2S3 and kNN-smoothing had better prediction accuracy than other imputation methods in restoring gene regulatory relationships across all GRN inference methods. These results have been added in lines 332-340 on page 16.

9. *In addition to AUPRC, AUROC is also commonly used for network prediction evaluation and thus is desired here.*

We used AUROC and AUPRC to evaluate the prediction accuracy in the revised manuscript. These results were described in response to comment 8 and can be found in lines 332-340 on page 16, Fig 6 and Fig S6.

10. *Abstract section. “novel” should be avoid used here, since the network learning algorithm, the major part of G2S3, is just adapted from Ref. 44. Lines 20-23: should be revised for “overall performance”.*

We replaced “novel” with “new”, and added “overall performance” in line 21 in Abstract.

11. *Introduction section. Line 88 “… is robust to outliers in the data” is not verified.*

We deleted this sentence in line 88 on page 5.

12. *Reference format should be carefully checked.*

We have carefully formatted all references following the journal requirements.

13. *Fig S3 seems almost repeated with Fig 1, only with additional SAUCIE.*

Yes, the two plots are the same except that Fig S3 includes SAUCIE. The gene-wise and cell-wise correlations of SAUCIE were much lower than that of the other methods, so that the boxplot of SAUCIE cannot be fully displayed on the current scale of Y axis. Therefore, we put the plot with SAUCIE in the supplement and put the same plot without SAUCIE in the main text.

14. *Fig S4. X-label, full text of “Widely (Mildly/Rarely) expressed genes” may be better.*

We relabeled Fig S4 accordingly.

15. *Fig 4 legend: “reversed graph embedding” is not clear. R packages or functions used for visualization here should be provided.*

Reversed graph embedding is a machine learning method to reconstruct complex single-cell trajectories in the R package Monocle 2. We added this information in line 656 on page 30.

16. *The phrase “recovering true gene expression” occurred many times throughout the manuscript but I don’t think this phrase is accurate. Not only the dropout but also the other expression values were globally changed after imputation (see Lines 520-521). So the recovered gene expression is not the true expression.*

We replaced “recovering true expression levels” with “recovering gene expression” throughout the revised manuscript.

17. *“cell lines” (Line 185 and other places) is not accurate. Cell line means in vitro cultured cells, but undifferentiated human ES (H1 and H9) should be from in vivo tissues.*

We replaced “cell lines” with “cells” in lines 183, 208 and 573.

18. *Page 20 Lines 421-422, “especially for genes with relatively low expression” seems a mistake expression? In addition, “G2S3 is the most computationally efficient…” seems not true, which is not reflected in Table S2.*

We deleted the sentence “especially for genes with relatively low expression”, and rephrased the sentence “G2S3 is the most computationally efficient” as “G2S3 is computationally efficient”.

19. *Page 29, “Improvement in …”, this is repeated with that in the Results section. I suggest to change such words in the methods section to, like, “Evaluation on …”.*

We deleted the words “Improvement in” in lines 648 and 658 on page 30.

20. *English should be improved. The text is not concise enough and sometimes not accessible. Grammar errors even still exist. Just to list a few: Fig 5 legend; Line 76; Lines 82-83; Line 296; Line 545. …*

We have fixed the listed grammar errors. We have also sent our manuscript to a professional text editor for grammar and language check throughout the revised manuscript.
